# Study on the Impact of Pole Spacing on Magnetic Flux Leakage Detection under Oversaturated Magnetization

**DOI:** 10.3390/s24165195

**Published:** 2024-08-11

**Authors:** Wenlong Liu, Lemei Ren, Guansan Tian

**Affiliations:** School of Thermal Engineering, Shandong Jianzhu University, Jinan 250100, China; 19861806215@163.com (W.L.); tgs4170@sdjzu.edu.cn (G.T.)

**Keywords:** magnetic flux leakage (MFL), leakage magnetic field, double peak–valley (DPV) phenomenon, magnetic pole spacing

## Abstract

Magnetic flux leakage (MFL) inspection employs leakage magnetic fields to effectively detect and locate pipeline defects. The spacing between magnetic poles significantly affects the leakage magnetic field strength. While most detectors typically opt for moderate pole spacing for routine detection, this study investigates the propagation characteristics of MFL signals at small pole spacings (under specimen oversaturated magnetization) and their impact on MFL detection. Through finite element simulation and experiments, it reveals a new signal phenomenon in the radial MFL signal *B_y_* at small pole spacings, the double peak–valley (DPV) phenomenon, characterized by outer and inner peaks and valleys. Theoretical analysis based on the simulation results elucidates the mechanisms for this DPV phenomenon. Based on this, the impact of defect size, pipe wall thickness, and magnetic pole and rigid brush height on MFL signals under small magnetic pole spacings is examined. It is demonstrated that, under a smaller magnetic pole spacing, a potent background magnetic field manifests in the air above the defect. This DPV phenomenon is generated by the magnetic diffusion and compression interactions between the background and defect leakage magnetic fields. Notably, the intensity of the background magnetic field can be mitigated by reducing the height of the rigid brush. In contrast, the pipe wall thickness and magnetic pole height exhibit a negligible influence on the DPV phenomenon. The emergence of the DPV precipitates a reduction in the peak-to-valley difference within the MFL signal, constricting the depth range of detectable defects. However, the presence of DPV increases the identification of defects with smaller opening sizes. These findings reveal the characterization of the MFL signal under small pole spacing, offering a preliminary study on identifying specific defects using unconventional signals. This study provides valuable guidance for MFL detection.

## 1. Introduction

Pipelines play a key role in oil and gas transmission. Their safe operation is crucial to energy support and economic development [1]. It is possible for these pipelines to develop cracks, corrosion, deformation, and other defects in the long-term transportation process, threatening the safety of production [2,3]. Therefore, it is necessary to test pipelines regularly. Nondestructive testing (NDT) is a widely used method for inspecting pipelines without causing damage to the tested material. The common NDT methods include ultrasonic testing (UT), eddy current testing (ECT), and magnetic flux leakage (MFL) testing [4]. Compared to other NDT methods, MFL detection technology has the advantages of a strong anti-interference ability, a fast signal acquisition speed, and no need for coupling agents; so, it has become the mainstream technology for pipeline detection worldwide [5,6].

Pipeline defects can be identified, located, and quantified by analyzing the MFL signals [7]. Many scholars have found that MFL signals are influenced by many factors, such as defect size, sensor detection angle and direction, temperature, stress, scanning speed, lift-off value, pipe shielding effects, and specimen surface roughness. Kopp et al. [8] employed a magnetic dipole model (MDM) to investigate how the defect size impacts MFL signals, establishing a clear correlation between signal characteristics and defect geometric parameters. Tri-axial signals were utilized to approximate defect opening sizes. To address probe tilt due to detector vibration during detection, Long et al. [9] proposed a dual-sensor probe to compensate for probe tilt. This approach effectively achieves the compensation of the original MFL signal. The scanning direction of the sensor also affects the MFL signal. Wu et al. [10] using the MDM revealed that the MFL signal is most effective when the defect’s extension direction is perpendicular to the scanning direction of the sensor. Wang et al. [11] developed a temperature-dependent MDM that considers the combined effects of the temperature and thermal stress, noting a parabolic signal amplitude variation within specific temperature ranges. Based on the J-A theory, Liu et al. [12] investigated the relationship between stress and the saturation magnetization strength of ferromagnets. They observed that, under high magnetic field stress, the saturation magnetization strength of ferromagnets decreases exponentially with the increase in stress. This reduction diminishes the amplitude of the MFL signal, potentially leading to an underestimation of defect depth. Wu investigated the impact of uniform and non-uniform stress on bi-axial MFL signals, observing changes in signal amplitude both perpendicular and parallel to the stress direction. Furthermore, it was noted that these signals effectively characterize the magnitude and distribution of stress inhomogeneity [13]. Eddy current effects, resulting from the relative motion between magnetization devices and pipelines, diminish MFL signal strength [14,15]. Pullen’s study revealed that increasing the detection speed results in insufficient magnetization strength in the pipe wall. This causes the MFL signal strength of outer wall defects to decrease, while that of inner wall defects increases [16,17]. Usarek found that both the tangential and normal components of the magnetic field increase linearly with speed. To compensate for this effect, an empirical fitting equation was employed to adjust the MFL signal [18]. The lift-off value between the sensor probe and the detection object increases the noise in the MFL signal. Peng et al. [19] and Feng et al. [20] analyzed the influence of the lift-off value on the MFL signal based on the MDM and finite element model (FEM). They proposed compensation methods for the sensor lift-off effect on the MFL signal. Liu et al. [21] addressed pipe wall shielding effects on MFL signal propagation, integrating a wall propagation compensation factor into the MDM for accurate defect detection on outer pipe walls. Deng et al. [22] discussed the effect of ferromagnetic specimen surface roughness on the MFL signals. It was found that surface roughness generates excess MFL signals, which could obscure weaker signals and increase the risk of missed defect detections. Additionally, Yousaf analyzed magnetic pole spacing impacts on the MFL testing of steel bars, determining the optimal spacing considering leakage magnetic field intensity and magnetic pole interaction effects [23].

In conclusion, while there have been many studies on the factors that affect MFL signals, research on the impact of magnetic pole spacing is still relatively scarce. This is because the majority of detectors have been designed with the pole spacing already set according to the conventional detection scenario. Generally, reducing the magnetic pole spacing can enhance the amplitude of the MFL signal. However, further reductions may disturb it [24]. Studies on the amplitude and waveform characteristics of MFL signals with small pole spacing are notably sparse, particularly when the pipe wall is oversaturated with magnetization.

This paper thoroughly investigates the variation pattern of MFL signals under small magnetic pole spacing through theoretical analysis and experimental research. Firstly, the influence of magnetic pole spacing on the MFL signal is analyzed, and a new signal phenomenon is discovered. The cause of the phenomenon is also analyzed through FEM simulations. Subsequently, we examine the effects of defect size, wall thickness, magnetic pole, and rigid brush height on the MFL signal under wall saturation and oversaturation conditions.

## 2. Methods

### 2.1. The Model of MFL Detection

**Figure 1:**
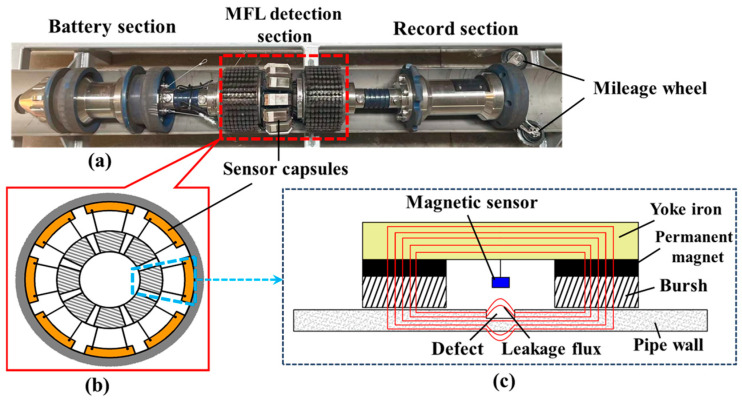
The principle of the MFL detection: (**a**) schematic diagram of MFL inspection detector, (**b**) cross-sectional illustration of MFL detection section, and (**c**) the principle of MFL signal collection.

The internal detector for the pipe MFL comprises a battery section, a detection section, a data recording section, and a mileage wheel, as illustrated in Figure 1a. Among these components, the MFL detection section is responsible for magnetizing the pipe wall and collecting the MFL signals using magnetic sensors. The collected signals are then filtered, amplified, and stored in the recording section. The battery section provides power to the detector and also drives the detector’s movement. The mileage wheel is capable of recording the detection mileage and movement speed of the detector. The magnetic sensors used to collect the MFL signals in the detection section are distributed as shown in Figure 1b. The sensor capsules are uniformly distributed on the whole circumference, and 12 Hall sensors are uniformly distributed in each sensor capsule.

Figure 1c illustrates the principle of MFL signal collection, which relies on the high magnetic permeability of ferromagnetic materials. To achieve a saturated or near-saturated state, a permanent magnet, a yoke iron, and a rigid brush are used to fully magnetize the pipe wall. In the absence of defects, the magnetic force lines run parallel to the inside of the pipe. However, if surface or near-surface defects, for example, are present, the magnetic force lines become obstructed, causing some of them to leak out of the pipe surface. This leakage magnetic field can be detected using a magnetic sensor, enabling the identification and characterization of the defect.

The magnetic field is the physical variable in the MFL detection system. Therefore, Maxwell equations are used to describe the leakage magnetic field. As this study does not consider the velocity effect, a static magnetic field model is used to describe the leakage magnetic field [25]. Thus, it can be described by

(1) ∇×H=J

(2) ∇·B=0

where ∇ is the Hami operator, *H* is the magnetic field strength, *J* is the equivalent current density, and *B* is the magnetic flux density.

The relationship between the electromagnetic materials is described as

(3) B=μ·H

where μ is the medium’s permeability.

Due to the source-free nature of magnetic fields, introducing the vector magnetic potential *A* allows for the following:

(4) ∇×A=B

From Equations (1)–(4) and the vector identity ∇ × (∇ × *A*) = ∇ (∇ *A*) − $\nabla^{2}$*A*, we can derive

(5) $$\frac{1}{\mu }\nabla^{2}\times A=-J$$

To solve Equation (5), the following boundary conditions are introduced:

(6) $$\left\{\begin{array}{c} A=0\;\;\;\;\;\;\;\;\;\;\mathrm{At}\;\mathrm{the}\;\mathrm{boundary}\;\mathrm{of}\;\mathrm{the}\;\mathrm{model}\\ A_{1}=A_{2}\;\;\mathrm{At}\;\mathrm{the}\;\mathrm{junction}\;\mathrm{of}\;\mathrm{two}\;\mathrm{mediums} \end{array}\right.$$

By utilizing the boundary conditions to solve Equation (5), the axial and radial components of the MFL signal can be calculated as follows:

(7) $$\left\{\begin{array}{c} B_{x}=\frac{\partial A}{\partial y}\;\;\;\;\\ B_{y}=-\frac{\partial A}{\partial x} \end{array}\right.$$

where *B_x_* and *B_y_* represent the axial and radial components of the magnetic field, B, respectively.

### 2.2. Finite Element Model of MFL Detection 

**Figure 2:**
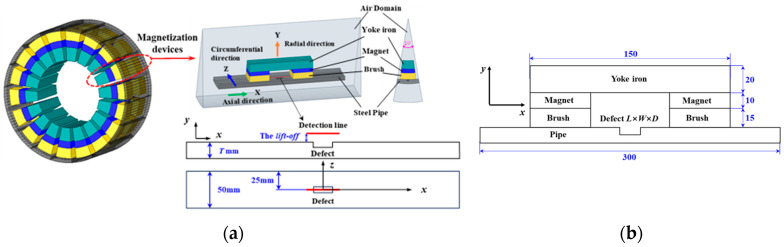
Three-dimensional defect leakage magnetic field FEM: (**a**) geometric model; (**b**) dimensions of the magnetizing device (mm).

**Figure 3:**
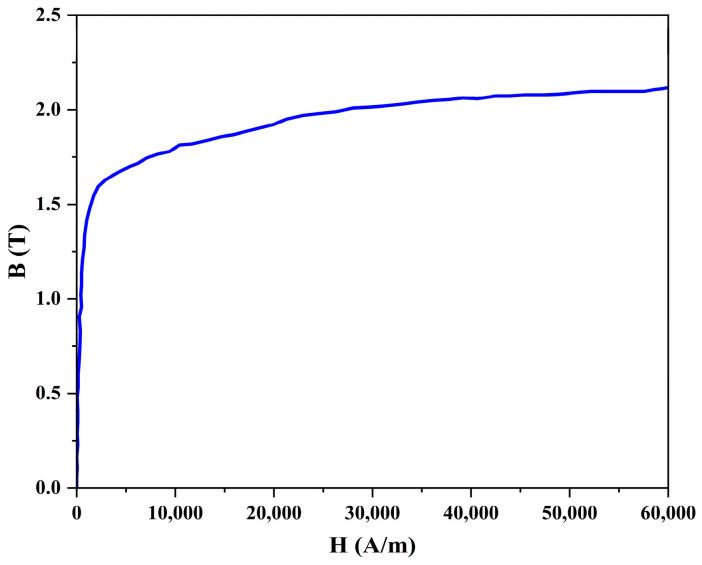
Nonlinear *BH* characteristic curve of the steel pipe.

The FEM is a numerical analysis model based on Maxwell equations for solving leakage magnetic fields and has found extensive application in the field of MFL inspection. This study utilized ANSYS 2021 R2 software to construct a complete 3D MFL detection FEM, as shown in Figure 2a. This consists of 18 magnetization device pieces, which are arranged in a circle around the pipe wall. The magnetization device comprises a yoke iron and two opposing permanent magnets. A layer of inflexible brushes is positioned on the surface of the permanent magnets, which come into direct contact with the steel pipe specimen. The inflexible brushes are magnetized to saturation by the permanent magnets, and their magnetic permeability can be considered constant. Their purpose is to prevent friction between the permanent magnets and the pipe wall during the detection process. The steel pipes used in high- and medium-pressure gas pipelines in urban areas have a diameter of 300 mm and a wall thickness T ranging from 6 mm to 10 mm. Figure 2b provides a detailed description of the composition and dimensions of the magnetization device, while Table 1 contains the material parameters. The pit-like defects on the inner surface of the steel pipe specimen have dimensions of length L × width W × depth D and are rectangular in shape. Figure 3 shows the nonlinear B-H curve of the steel pipe. The air domain encloses the entire MFL detection device.

For the convenience of data extraction, the detection lines were arranged axially above the defects, as indicated by the red line in Figure 2a. The lift-off value was set at 1 mm, and the sampling interval was set to 0.001 mm.

Mesh independence validation: To account for the range of cases with larger specimen volumes and small defect sizes, various grid sizes are utilized in different regions [26]. The computational domain is partitioned into an unstructured mesh, with local refinement applied to the air domain near the defect. As the mesh is progressively refined, the computed results tend to become more stable. A mesh count of 123,000 was adopted in this study as further increasing the number of meshes does not yield substantial changes in the computed results.

### 2.3. Physical Experiments

**Figure 4:**
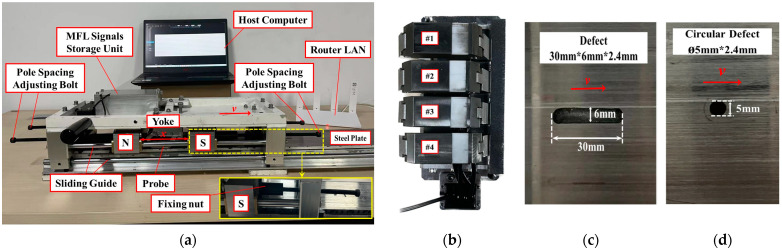
The experimental setup: (**a**) MFL detection device and signal transmission processing system, (**b**) schematic diagram of sensor probe, (**c**) rectangular metal loss defect, and (**d**) circular metal loss defect.

The manual adjustment of the pole spacing is challenging due to the strong magnetic suction in front of the magnetic poles and the yoke. Consequently, the research team devised a special mechanical structure to design an MFL detection device that employs bolts to facilitate the autonomous adjustment of the pole spacing X, as illustrated in Figure 4a. The fixing nut is fixed on the magnetic poles, and by adjusting the bolt during the experiment, the magnetic poles can be driven to move along the slide until the ideal pole spacing X is reached. The magnetizing device of the MFL detection system comprises a yoke and poles, which are connected to the rigid brushes that magnetize the tube wall. The magnetic poles have a remanent magnetic strength of 1.3 T. The MFL detection probe comprises four Hall sensors, with an axial sampling interval of 0.05 mm and a circumferential sampling interval of 5 mm, as shown in Figure 4b. The scanning speed is 0.1 m/s, and the sensor lift-off value is set to 2 mm. The flat-plate ferromagnetic specimen being tested has a wall thickness of 6 mm. A rectangular metal defect with dimensions of *L* = 30 mm, *W* = 6 mm, and *D* = 2.4 mm, along with a circular metal loss defect with dimensions of *Ø* = 5 mm and *D* = 2.4 mm, were observed on the specimen, as shown in Figure 4c,d. The MFL signals collected by the sensors are initially stored in the signal storage device of the testing device. After detection, they are transmitted to the host computer for data processing and analysis using the local area network (LAN) provided by the router. The detection principle, material properties, and probe performance of this experimental setup are identical to those of a real pipe MFL internal detector. Consequently, this detector can be employed as a highly accurate substitute in theoretical analyses of the impact of magnetic pole spacing on the MFL signal.

## 3. Results of Simulation and Experiments

### 3.1. FEM Simulation

#### 3.1.1. For Rectangular Defects

**Figure 5:**
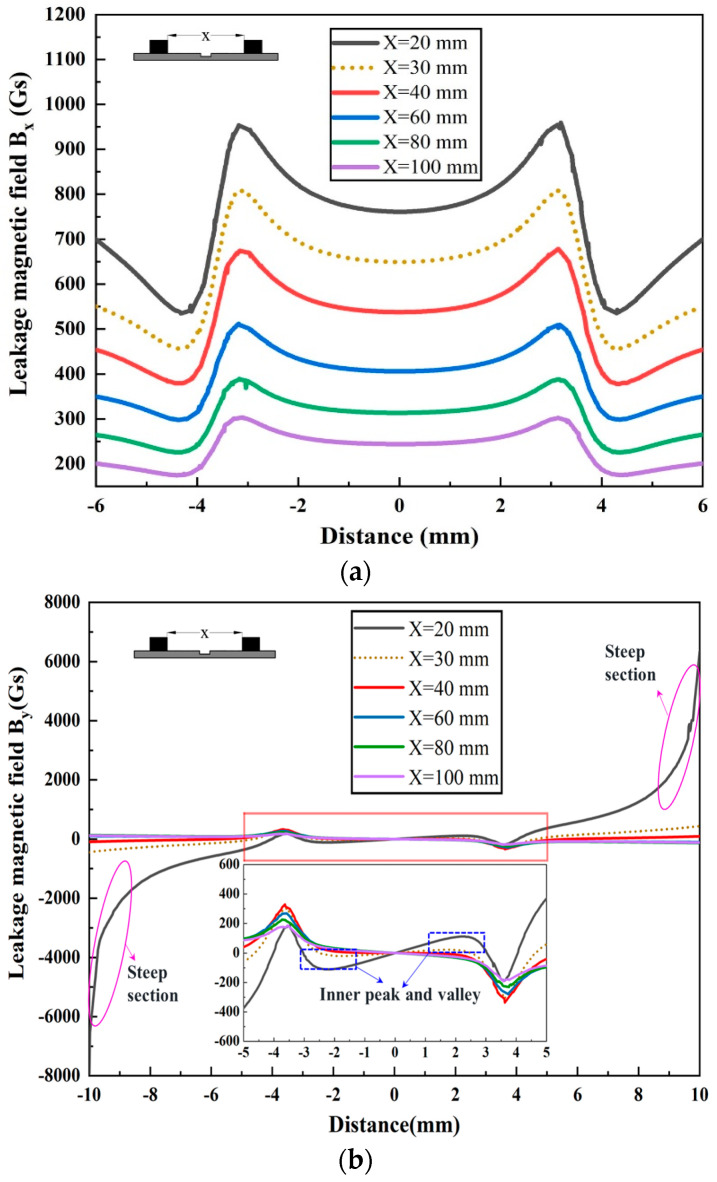
Propagation characteristics of the MFL signal with different magnetic pole spacings: (**a**) the leakage magnetic field axial component *B_x_*; (**b**) the leakage magnetic field radial component *B_y_*.

Rectangular defects are frequently studied in the context of MFL detection. When analyzing the characteristics of these defects, they can generally be approximated as rectangular by using three equivalent shape parameters: equivalent length, width, and depth [27,28]. Figure 5 shows the variation in the MFL signal for a defect with dimensions of *L* = 6 mm, *W* = 3 mm, and *D* = 5 mm at five different magnetic pole spacings *X* (20 mm, 40 mm, 60 mm, 80 mm, and 100 mm), with a wall thickness of *T* = 10 mm. The magnetic field *B_x_* is illustrated in Figure 5a. The graph shows that the magnetic field *B_x_* is enhanced as the magnetic pole spacing decreases. It is suggested that decreasing the magnetic pole spacing improves the detection accuracy. Additionally, the amplitude of the magnetic field *B_x_* increases at a greater rate when the pole spacing is reduced from 100 mm to 40 mm. The simulation results demonstrate that reducing the magnetic pole spacing increases the amplitude of *B_x_* while maintaining its waveform. Conversely, Figure 5b illustrates significant alterations in both the waveform and amplitude of the magnetic field *B_y_*. Hence, due to spatial limitations, this paper mainly concentrated on analyzing the magnetic field *B_y_* rather than *B_x_*.

**Figure 6:**
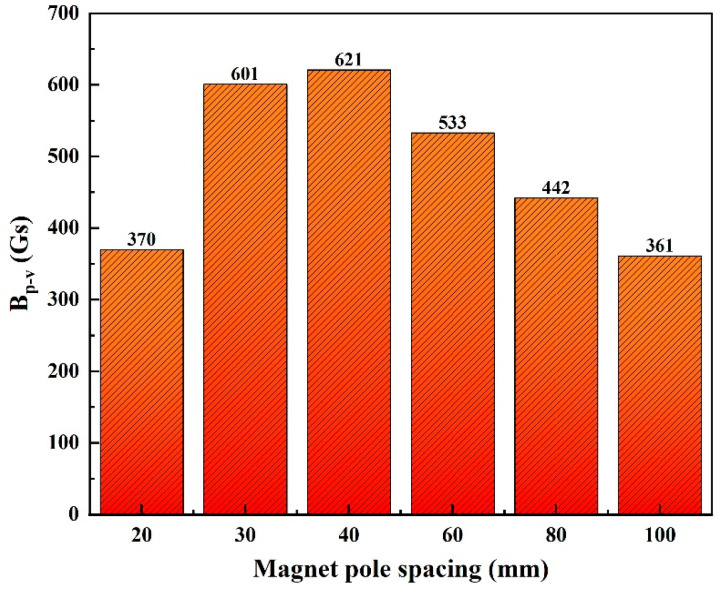
Column chart of the *B_p-v_* of the magnetic field *B_y_*.

When the pole spacing is 20 mm, *B_y_* demonstrates a distinct double peak and valley pattern. The outer peak and valley exhibit positive values followed by negative ones, whereas the inner peak and valley demonstrate negative values followed by positive ones. Additionally, the MFL signal curve at both ends of the scanning range displays a steep slope, with the signal intensity rapidly increasing. This study refers to this phenomenon as a double peak–valley (DPV). Even with a magnetic pole spacing of 30 mm, a slight DPV phenomenon is observed in *B_y_*. This phenomenon results in a decrease in the *B_p-v_* (the peak-to-valley value) of the *B_y_* signal when the magnetic pole spacing is reduced from 40 mm to 20 mm. Figure 6 displays the histogram of the *B_p-v_* of *B_y_*. The figure shows that the *B_p-v_* of the MFL signal first increases and then decreases as the magnetic pole spacing decreases. At a magnetic pole spacing of 40 mm, *B_p-v_* reaches a maximum value of 621 Gs. However, at a magnetic pole spacing of 20 mm, the *B_p-v_* of the MFL signal is only 370 Gs. At this time, the pipe wall is oversaturated with magnetization, which disrupts the distribution of the magnetic field and hampers the detection of defects in the pipe. Therefore, this paper assumed that, in the FEM simulation, the pipe wall can be considered as having oversaturated magnetization when the magnetic pole spacing is less than or equal to 20 mm.

**Figure 7:**
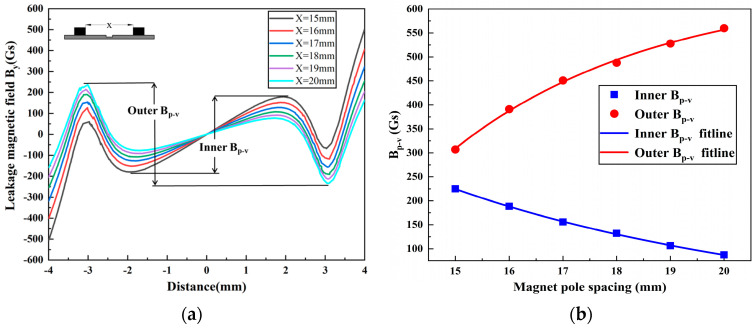
The MFL signals with different magnetic pole spacings: (**a**) the leakage magnetic field *B_y_*; (**b**) variation in *B_p-v_*.

To investigate the effect of smaller magnetic pole spacings on the MFL signals of pipeline defects, this study conducted simulations and calculations for magnetic pole spacings ranging from 15 mm to 20 mm. The magnetic field *B_y_* is shown in Figure 7. With the decrease in magnetic pole spacing, the DPV phenomenon becomes more pronounced. Concurrently, the inner *B_p-v_* steadily increases while the outer *B_p-v_* decreases, and the rates of their increase and decrease also accelerate progressively.

#### 3.1.2. For Circular Defects

**Figure 8:**
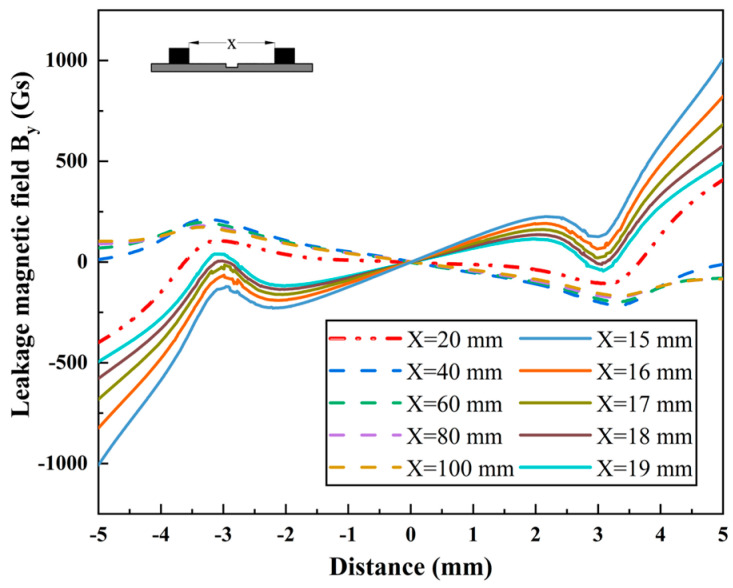
The variation in the leakage magnetic field *B_y_* of circular defect with different pole spacings.

Given the varied contours of actual pipe defects, this paper also investigated circular defects. The research team employed FEM to compute circular defects with an opening radius of 3 mm and a depth of 5 mm across various pole spacings, with a wall thickness of *T* = 10 mm. As depicted in Figure 8, the amplitude of the MFL signal reaches its maximum at a pole spacing of 40 mm. When the magnetic pole spacing is less than 20 mm, the MFL signals begin to exhibit DPV phenomena, with sharp increases in the signal amplitude at both ends of the sweep range. As the magnetic pole spacing decreases, the DPV signature of the signal becomes apparent. Compared to the rectangular defects illustrated in Figure 7a, both demonstrate similar characteristic patterns of change.

### 3.2. Physical Experiments

**Figure 9:**
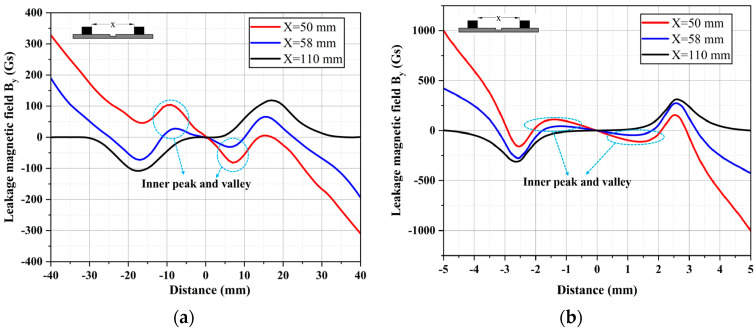
The leakage magnetic field *B_y_* at the magnetic pole spacings of 50 mm, 58 mm, and 110 mm: (**a**) rectangular metal loss defect; (**b**) circular metal loss defect.

The research team conducted experiments to test the defect MFL signals at the magnetic pole spacings of 50 mm, 58 mm, and 110 mm using the experimental setup and defect samples shown in Figure 4. The magnetic field *B_y_* is presented in Figure 9. For both rectangular and circular metal loss defects, at a pole spacing of 110 mm, a typical radial MFL signal is observed, characterized by a pair of peaks and valleys corresponding to the axial length of the defect. However, with a reduced magnetic pole spacing of 58 mm, the magnetic field *B_y_* begins to exhibit DPV phenomena. As the magnetic pole spacing further decreases, the outer *B_p-v_* decreases continuously while the inner *B_p-v_* increases steadily. Simultaneously, the amplitude of *B_y_* also increases sharply on both sides of the scanning range, consistent with the distribution characteristics of simulated signals in Figure 7 and Figure 8, and the variation in *B_p-v_* with magnetic pole spacing. Moreover, the experimental signal amplitudes remain within the same order of magnitude as the simulated signals, further confirming the presence of DPV and the correctness of the FEM.

Additionally, it is noteworthy that, under the same magnetic pole spacing, the DPV phenomenon exhibited by the rectangular defect in Figure 9a is significantly more pronounced than that of the circular defect in Figure 9b. This difference arises from the shorter axial length of the circular defect compared to the rectangular one, resulting in a less pronounced DPV in their radial MFL signals. The influence of defect size on the DPV phenomenon will be further discussed in Section 4.

## 4. Discussion

This section compares and investigates the effects of defect size, pipe wall thickness, magnetic pole, and rigid brush height on the MFL signal in the case of saturated and oversaturated magnetization (selected pole spacings of 40 mm and 20 mm, respectively), using a rectangular defect as an example. The mechanisms for the DPV phenomenon of the MFL signal are also theoretically analyzed based on the FEM simulation results.

### 4.1. The Effect of Defect Depth on the Characteristics of MFL Signals

**Figure 10:**
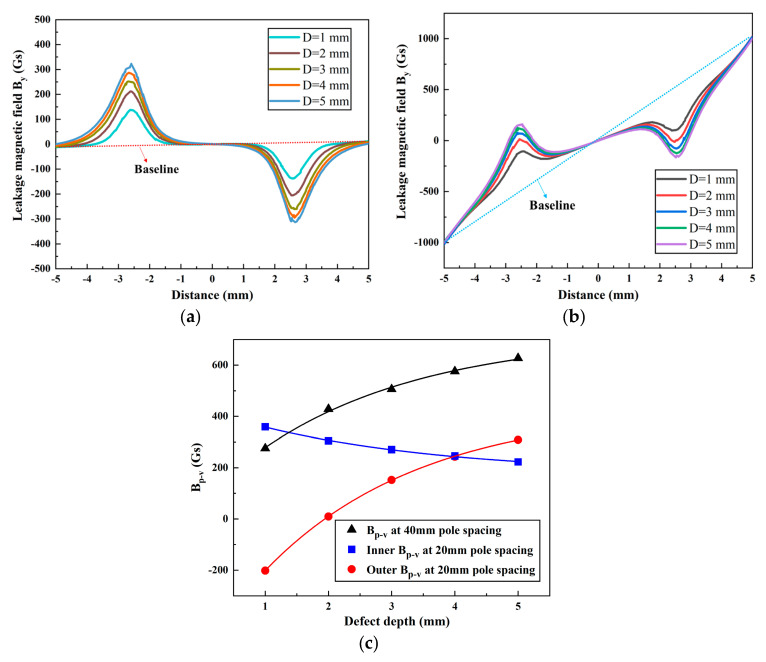
The leakage magnetic field *B_y_* at different defect depths: magnetic pole spacings of (**a**) 40 mm and (**b**) 20 mm; and (**c**) the variation in *B_p-v_*.

A pipe wall thickness of *T* = 10 mm and a defect length and width of *L* = 5 mm and *W* = 3 mm, respectively, were used for the simulation calculations. The magnetic field *B_y_* was simulated for defect depths *D* = 1, 2, 3, 4, and 5 mm at magnetic pole spacings of 40 mm and 20 mm, as shown in Figure 10a,b, respectively. Figure 10c shows the variation curve of the *B_p-v_* of the radial MFL signal *B_y_* concerning the defect depth for different magnetic pole spacings.

From the figure, it can be seen that, for a magnetic pole spacing of 40 mm, the *B_p-v_* shows an increasing trend as the defect depth ranges from 1 mm to 5 mm. Specifically, the *B_p-v_* increases from 275 Gs to 627 Gs with a gradually decreasing growth rate. At a magnetic pole spacing of 20 mm, as the defect depth increases from 1 mm to 5 mm, the outer *B_p-v_* gradually increases and reaches a value of 510 Gs. Conversely, the inner *B_p-v_* gradually decreases to a value of 136 Gs. When the defect depth is less than 4 mm, the inner *B_p-v_* is higher than that on the outer one. However, when the defect depth is between 4 and 5 mm, the outer *B_p-v_* becomes higher than that of the inner one. Furthermore, at a magnetic pole spacing of 20 mm, the MFL curve shows abrupt changes at both ends of the scan. The baseline of the MFL signal changes from a horizontal to an inclined line, as shown by the dashed line in the figure.

Comparing the MFL signals under two different magnetic pole spacings, it was observed that, for defect depths ranging from 1 mm to 5 mm, the *B_p-v_* under 40 mm magnetic pole spacing showed an average increase of 380 Gs compared to that under 20 mm. This indicates that, as the distance decreases from 40 mm to 20 mm, the intensity of the MFL signal decreases significantly. In addition, the outer *B_p-v_* at the 20 mm pitch and 5 mm defect depth is very similar to the *B_p-v_* at the 40 mm pitch and 1 mm defect depth. The presence of DPV may result in an underestimation of defect depth and a narrower range of detectable defect depths.

### 4.2. The Effect of Defect Length on the Characteristics of MFL Signals

**Figure 11:**
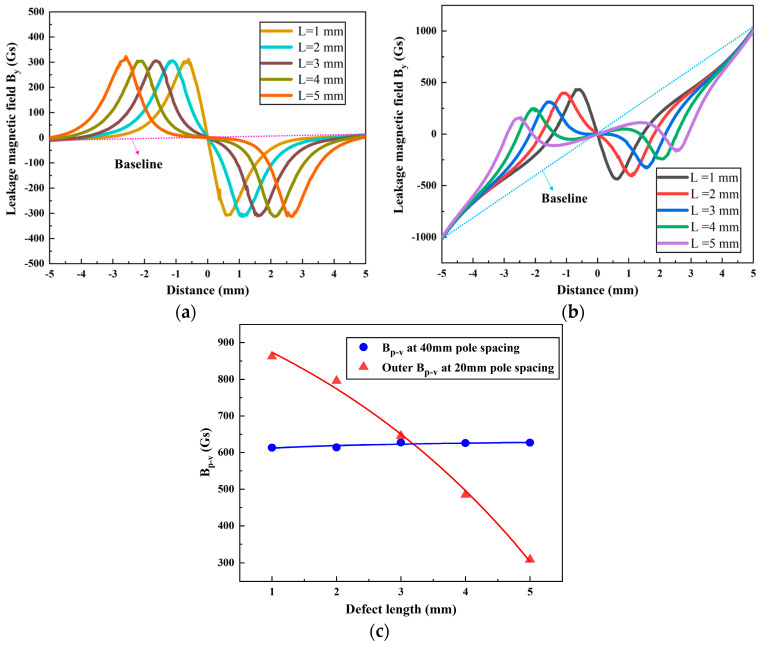
Leakage magnetic field *B_y_* at different defect lengths: magnetic pole spacings of (**a**) 40 mm and (**b**) 20 mm, and (**c**) the variation in *B_p-v_*.

A pipe wall thickness of *T* = 10 mm and a defect depth and width of *D* = 5 mm and *W* = 3 mm, respectively, were used for the simulation calculations. The magnetic field *B_y_* was simulated for defect lengths *L* = 1, 2, 3, 4, and 5 mm at the magnetic pole spacings of 40 mm and 20 mm, as shown in Figure 11a,b, respectively. Figure 11c shows the variation in the *B_p-v_* of the magnetic field *B_y_* concerning the defect length for different magnetic pole spacings. 

Figure 11 show that, at a magnetic pole spacing of 40 mm, increasing the defect length from 1 mm to 5 mm results in an expansion of the horizontal distance between peaks and valleys (corresponding to the defect length), while *B_p-v_* remains relatively constant. For a magnetic pole spacing of 20 mm, the outer *B_p-v_* decreases by 553 Gs as the defect length increases from 1 mm to 5 mm. The baseline of the MFL signal curve changes from horizontal to inclined, as indicated by the dashed lines in the figure. It is evident that, for a magnetic pole spacing of 40 mm, the defect length has a minimal effect on the intensity of the magnetic field, *B_y_*. However, when the magnetic pole spacing is reduced to 20 mm, the intensity of the magnetic field, *B_y_*, decreases rapidly as the defect length increases.

From Figure 11c, it can be seen that, in the range of defect lengths from 1 mm to 3 mm, the outer *B_p-v_* for a magnetic pole spacing of 20 mm is greater than that for 40 mm. However, as the defect length exceeds 3 mm, the *B_p-v_* for a magnetic pole spacing of 40 mm exceeds that for 20 mm. From this, it can be seen that the MFL signal is more stable at a spacing of 40 mm, allowing a wider range of defect lengths to be detected. Conversely, a spacing of 20 mm is more suitable for detecting smaller defects. Therefore, the presence of DPV facilitates the detection of defects with smaller opening dimensions. 

**Figure 12:**
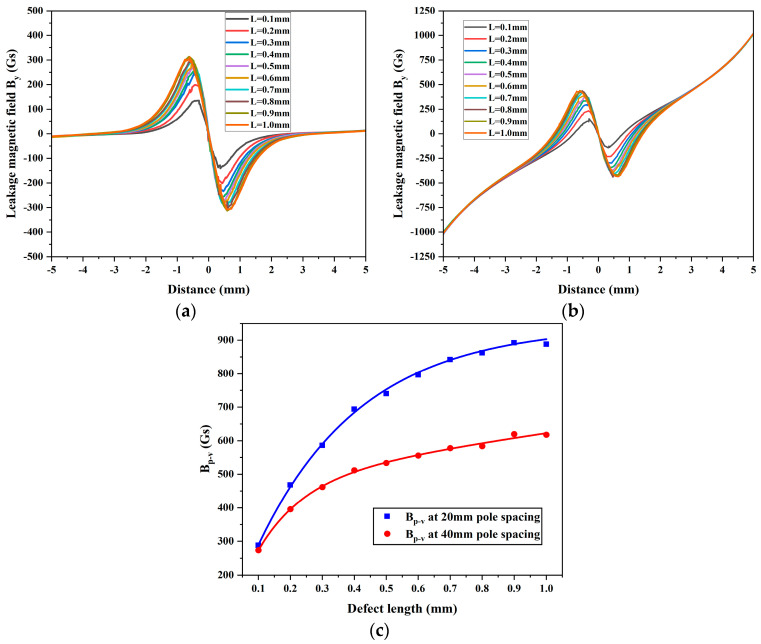
Leakage magnetic field *B_y_* at smaller defect lengths: magnetic pole spacings of (**a**) 40 mm and (**b**) 20 mm, and (**c**) the variation in *B_p-v_*.

Based on the above discussion, this study also discussed the detection range of small defect sizes using DPV signals. Magnetic field *B_y_* was simulated for defect lengths ranging from 0.1 mm to 1 mm in steps of 0.1 mm, at magnetic pole spacings of 40 mm and 20 mm, illustrated in Figure 12a,b, respectively. Figure 12c demonstrates the variation in *B_p-v_* of magnetic field *B_y_* with respect to the defect length. The results indicate that, within the range of defect lengths from 0.1 mm to 1 mm, the *B_p-v_* of DPV signals at a 20 mm pole spacing consistently exceeds that at a 40 mm pole spacing. Therefore, in conjunction with the findings from Figure 11, this study suggests that DPV signals, compared to conventional MFL signals, are more advantageous in identifying microdefects with lengths smaller than 3 mm. In the future, this specific signal may be utilized for detecting certain unique sizes.

### 4.3. The Effect of Wall Thickness on the Characteristics of MFL Signals

**Figure 13:**
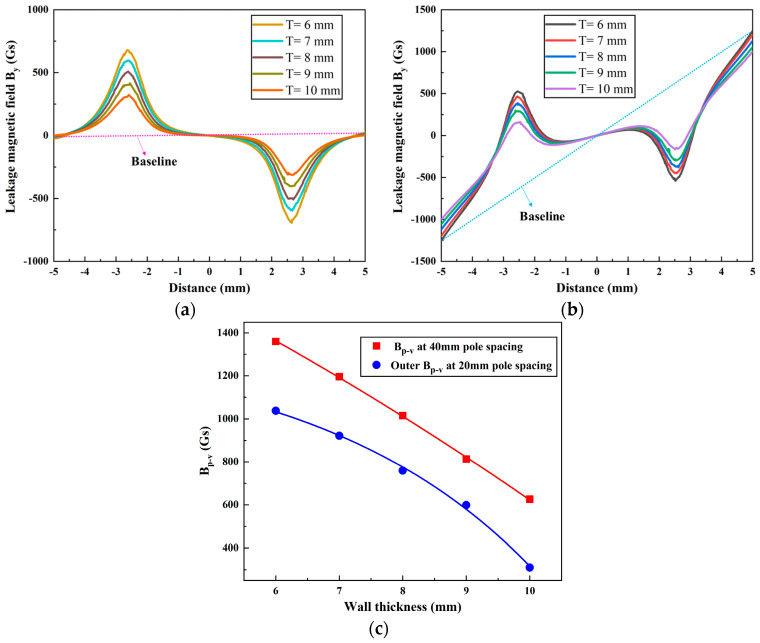
The leakage magnetic field *B_y_* at different wall thicknesses: magnetic pole spacing of (**a**) 40 mm and (**b**) 20 mm, and (**c**) the variation in *B_p-v_*.

For a pipe defect of length *L* = 5 mm, width *W* = 3 mm, and depth *D* = 5 mm, the corresponding magnetic field *B_y_* was simulated and calculated for wall thicknesses *T* = 6, 7, 8, 9, and 10 mm at magnetic pole spacings of 20 mm and 40 mm. The corresponding results are shown in Figure 13a,b, respectively. In addition, Figure 13c shows the variation in the *B_p-v_* of the magnetic field *B_y_* as a function of the pipe wall thickness for different magnetic pole spacings.

As shown in Figure 13, when the pipe wall thickness increases from 6 mm to 10 mm, the average *B_p-v_* at a spacing of 40 mm is approximately 276 Gs higher than that at 20 mm. Additionally, at 40 mm, *B_p-v_* decreases by 734 Gs. At 20 mm, the outer *B_p-v_* decreases by 734 Gs. It is noteworthy that the reduction in signal intensity appears to be similar regardless of the magnetic pole spacing used. In conclusion, increasing the wall thickness has a negative effect on MFL detection and has a similar effect on the MFL signal at different pole spacings.

### 4.4. The Effect of the Geometrical Height of the Magnetic Pole and Rigid Brush on the Characteristics of MFL Signals

**Figure 14:**
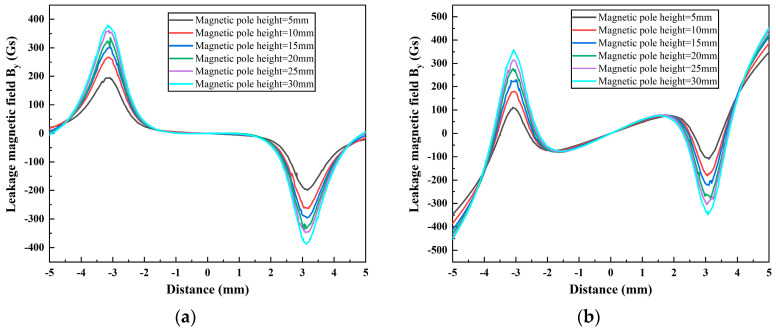
The leakage magnetic field *B_y_* at different magnetic pole heights, with magnetic pole spacings of (**a**) 40 mm and (**b**) 20 mm.

**Figure 15:**
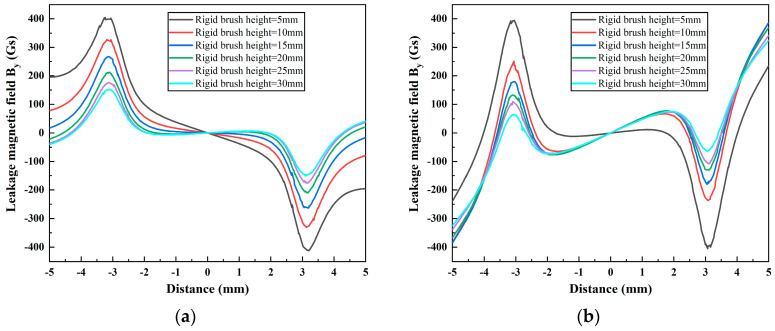
The leakage magnetic field *B_y_* at different rigid brush heights, with magnetic pole spacings of (**a**) 40 mm and (**b**) 20 mm.

In this section, the effects of the height of the magnetic pole (5 mm, 10 mm, 15 mm, 20 mm, 25 mm, and 30 mm) and rigid brush (5 mm, 10 mm, 15 mm, 20 mm, 25 mm, and 30 mm) on the MFL signals with different pole spacings are investigated using FEM simulations. The results are shown in Figure 14 and Figure 15, respectively.

As illustrated in Figure 14a,b, the amplitude of the MFL signal at the two pole spacings appears to increase with the magnetic pole height. This is due to an increase in the size and magnetizing ability of the magnet, which leads to an increase in the strength of the leakage magnetic field. As indicated by the results for the pole spacing of 20 mm, the magnetic pole height does not have a significant effect on the magnitude of the DPV signal.

Figure 15a,b illustrate that the MFL signal amplitude tends to increase at both pole spacings as the rigid brush height decreases. This is due to the fact that a reduction in the height of the rigid brushes results in a reduction in the magnetic flux required to magnetize the rigid brushes, while simultaneously reducing the leakage flux of the rigid brushes in the magnetic circuit. This, in turn, increases the magnetic flux in the wall of the ferromagnetic tube and enhances the strength of the leakage magnetic field. At a pole spacing of 20 mm, the reduction in the rigid brush height weakens the characteristics of the DPV signal. In particular, when the rigid brush height is reduced to 5 mm, the DPV phenomenon almost disappears, and the degree of sudden change in the signal amplitude at the two ends of the sweep range is also reduced. This indicates that the height of the rigid brush can be reduced to diminish the DPV of the MFL signal. The rationale behind this phenomenon will be elucidated in greater detail in Section 4.5.

### 4.5. Theoretical Analysis of the Mechanisms of DPV Phenomena

**Figure 16:**
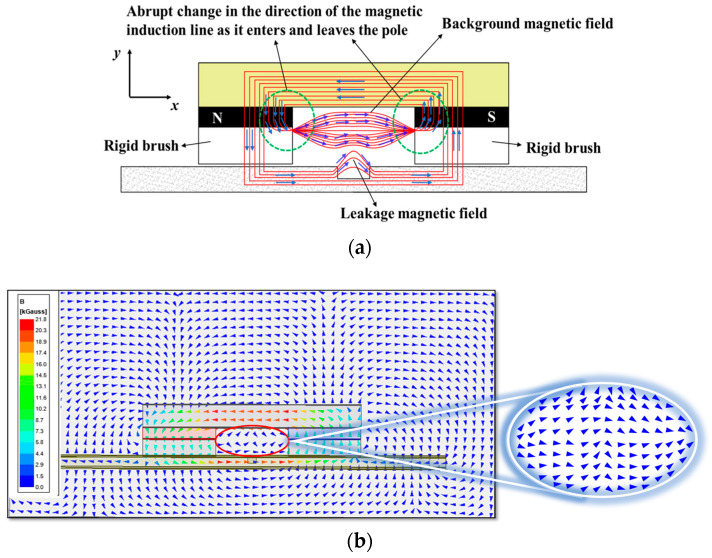
Schematic diagrams of the background magnetic field and defect leakage magnetic field: (**a**) magnetic field line diagram; (**b**) magnetic field vector diagram.

It is worth noting that, in both the experiments and FEM simulations, the magnetic field *B_y_* curves at the ends of the scan range become even steeper as the magnetic pole spacing decreases. In some cases, the difference in signal values between the two ends exceeds the inner and outer *B_p-v_*. This can cause the peak-to-valley difference in the MFL signals of the pipe defects to be overshadowed by the MFL signal curves at the ends of the scan range during actual inspection scenarios. As a result, it becomes difficult to distinguish the MFL signals associated with pipe defects. This scarcity is primarily attributed to the presence of significant background magnetic fields. The interaction between the magnetic poles generates a background magnetic field above the defect [29]. As the magnetic field lines move away from the north pole, they exhibit closed-loop behavior and enter the south pole, causing a change in direction as they leave and enter the poles. See Figure 16a for a detailed illustration of this phenomenon. As the magnetic field lines approach the magnetic pole, they tend to converge towards it, resulting in an increased density of field lines. This ultimately results in a higher magnetic field intensity in the vicinity of the magnetic pole. As shown in Figure 16b, the FEM simulation provides clear evidence of the magnetic field distribution between the two magnetic poles and further confirms the previous conclusions.

In general, the scanning range selected by the sensor is much smaller than the distance between the magnetic poles, which means that there is only a relatively stable background magnetic field within the scanning range. However, if the distance between the magnetic poles is too small, the detection area above the defect becomes very limited due to the restricted space. As a result, the sensor may collect magnetic field signals from the vicinity of the magnetic pole, where the direction and intensity of the magnetic field lines change abruptly within the background magnetic field. 

**Figure 17:**
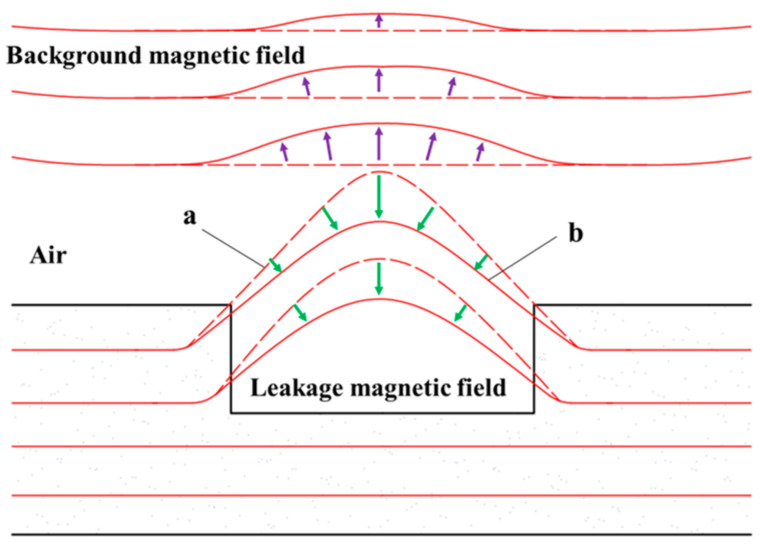
Schematic diagram of the magnetic compression effect.

The DPV phenomenon in the magnetic field *B_y_* is also attributed to the background magnetic field. As the magnetic pole spacing decreases, a narrow detection region is formed due to the presence of a strong background magnetic field. When the background magnetic field is aligned parallel to the leakage magnetic field, their magnetic fields impede each other’s diffusion, resulting in a magnetic compression effect [29]. As shown in Figure 17, this effect compresses the leakage field in the air from distribution ‘a’ to ‘b’, resulting in a decrease in the intensity of the leakage magnetic field. As the magnetic pole spacing decreases, the detection area encounters an uneven distribution of MFL signals due to the influence of the magnetic compression effect [24]. Consequently, multiple directional MFL signals overlap, ultimately leading to the formation of DPV. 

**Figure 18:**
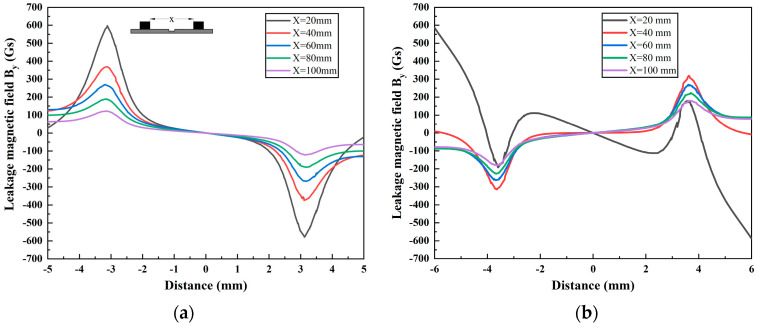
Leakage magnetic field variation at different pole spacings: (**a**) shielded background magnetic field; (**b**) altered pole directions.

Based on the above findings, the presence of a background magnetic field significantly influences the DPV phenomenon. Therefore, this paper further investigated the effects of shielding the background magnetic field on this phenomenon. As documented in the literature [30], when a ferromagnetic material is magnetized, a magnetic flux leaks from the ferromagnetic material into the air, which is known as refractive flux. However, this leakage can be mitigated by employing a closed magnetic circuit. The simulation results depicted in Figure 16 illustrate that, within the MFL detection device, the background magnetic field is mainly formed by the refraction of the leakage flux from the rigid brushes on both sides to the air domain above the defect; that is, the magnetic circuit forms an open circuit in the rigid brush part. Building upon this analysis, in this section, the height of the rigid brushes is set to zero and the ferromagnetic pipe wall is magnetized directly using the magnetizing device (yoke and poles) to establish a closed magnetic circuit, thereby eliminating the background magnetic field. The subsequent verification utilizing FEM, as shown in Figure 18a, demonstrates that, upon removing the background magnetic field generated by the rigid brush, the MFL signal’s amplitude increases solely with the reduction in magnetic pole spacing, and there is no DPV phenomenon or sharp increase in the signal amplitude at either end of the sweep range. This suggests that the background magnetic field is the direct cause of the DPV phenomenon observed in the MFL signal.

Additionally, this paper explores the influence of the direction of the background magnetic field on the DPV phenomenon. We reversed the N and S pole directions of the two magnetic poles in the FEM depicted in Figure 2a and simulated the MFL signals for magnetic pole spacings of 20 mm, 40 mm, 60 mm, 80 mm, and 100 mm. The simulation results are illustrated in Figure 18b. Even when the pole spacing is 20 mm, the MFL signal exhibits a clear DPV phenomenon. In comparison with Figure 5b, the amplitude of the MFL signal remains largely unchanged; only the signal direction varies.

## 5. Conclusions

This study delves into the impact of smaller magnetic pole spacings on the propagation characteristics of MFL signals using a leakage magnetic field FEM and a physical experiment. Moreover, the effects of defect size, pipeline wall thickness, and magnetic pole and rigid brush height on the MFL signals are also investigated under smaller pole spacings. The following conclusions are drawn:

With the reduction in magnetic pole spacing, a novel signal phenomenon emerges within the radial MFL signal. This phenomenon, termed the DPV, is distinguished by an outer peak and valley enclosing an inner peak and valley. Moreover, the signal amplitude at both ends of the sweep range also exhibits a sharp increase. The formation of this phenomenon can be attributed to the magnetic diffusion and compression interactions between the background magnetic field and the defect leakage magnetic field. The background magnetic field is primarily generated by the leakage magnetic flux of the rigid brush. By reducing the height of the rigid brush, the leakage magnetic flux from the rigid brushes can be diminished, thereby reducing the intensity of the background magnetic field and ultimately eliminating the DPV phenomenon. However, neither the pipe wall thickness nor the magnetic pole height exhibits a significant influence on the DPV phenomenon within the MFL signal. In comparison to the MFL signal under conventional magnetic pole spacing, the DPV signal at small pole spacing diminishes the detectable depth range of defects but enhances the identification rate of defects with small opening dimensions.

## Figures and Tables

**Table 1:** Material parameter settings.

Components	Materials	Permeability	Remanent Magnetization (T)
Magnet	Neodymium	1	1.3
Magnetizer	Iron	5000	-
Brush	Steel 1008	3000	-
Steel Pipe	Steel	*B-H* curve in Figure 3	-
Air Domain	Air	1	-

## Data Availability

Due to the confidentiality requirements of the project responsible unit, the data provided in this study should be provided at the request of the corresponding author, because the project has not yet been concluded.
